# Blood Biochemical and Hematological Study after Subacute Intravenous Injection of Gold and Silver Nanoparticles and Coadministered Gold and Silver Nanoparticles of Similar Sizes

**DOI:** 10.1155/2018/8460910

**Published:** 2018-07-22

**Authors:** Ji Hyun Lee, Mary Gulumian, Elaine M. Faustman, Tomomi Workman, KiSoo Jeon, Il Je Yu

**Affiliations:** ^1^Institute for Risk Analysis and Risk Communication, Department of Environmental and Occupational Health Sciences, University of Washington, Seattle, WA, USA; ^2^National Institute for Occupational Health, and Hematology and Molecular Medicine Department, University of the Witwatersrand, Johannesburg, South Africa; ^3^HCTm, Co. Ltd., Icheon, Republic of Korea

## Abstract

**Background:**

To investigate the effect of subacute intravenous administration AgNP (silver nanoparticles, 10 nm) and AuNP (gold nanoparticles, 12.8 nm) and AgNP/AuNP mixture to blood biochemistry, hematology, and platelet coagulation, subacute toxicity study was conducted.

**Methods:**

AuNP and AgNP in which their size distribution was not statistically different, mixed or separate, were injected into the caudal vein of male Sprague-Dawley rats for 4 weeks. The rats were allowed to recover for a further 4 weeks in order to examine systemic toxicity expressed in the blood biochemistry and hematology. The dose groups (5 males per group for the administration and 3 males for the recovery) consisted of 7 divisions, i.e., control, AgNP (with a low dose of 10 *μ*g/kg/day and a high dose of 100 *μ*g/kg/day), AuNP (with a low dose of 10 *μ*g/kg/day and a high dose of 100 *μ*g/kg/day), and mixed AgNP/AuNP (with a low dose of 10/10 *μ*g/kg/day and a high dose of 100/100 *μ*g/kg/day).

**Results:**

There were no significant dose-related changes in the hematology and blood biochemical values for the rats. Coagulation time in terms of the active partial thromboplastin time (APTT) and prothrombin time (PT) did not show any significant changes, when compared to the control group.

**Conclusion:**

The subacute injection of AuNP and AgNP or their mixture did not induce any noticeable systemic toxicity.

## 1. Introduction

The fast-growing number of applications of manufactured nanomaterials in nanomedicine area such as drug delivery systems, medical devices, nanodrug, and nanoenabled medicinal products is expected to increase significantly. In biomedical applications, AuNPs (gold nanoparticles) are applied as potential carriers for drug delivery, imaging molecules, and even genes [1] and for the development of novel cancer therapy products [2]. Nanosilver or silver nanoparticles (AgNP) for their effective antimicrobial properties have been used in many biomedical and consumer products. Safety of these nanoparticles has been tested via many routes of administration such as oral, inhalation, dermal, intravenous, etc., and from short- to long-term durations. For these nanoparticles, intravenous injection of these nanoparticles would be an essential process to use for biomedical applications. The toxicokinetics of AgNPs including tissue distribution studies have been already conducted [3–6]. However, all these safety and toxicokinetic studies have been always conducted only for exposure to single type of nanoparticles and not for coexposure of different types of nanoparticles.

We have previously studied such coexposures where AuNP (12.8 nm) and AgNP (10 nm) were injected, mixed or separate, into the caudal vein of male Sprague-Dawley rats for 4 weeks. The rats were allowed to recover for a further 4 weeks in order to examine the differences in AuNP / AgNP tissue distribution and clearance. As a result, AgNP and AuNP showed different toxicokinetic properties and the mixed administration of AgNP with AuNP resulted in mutual reduction of their tissue distribution which appeared to be due to competitive inhibition. Furthermore, this subacute intravenous injection study has suggested that these nanoparticles were distributed to the organs in particulate instead of ionic forms [7]. These subacute intravenous injection studies also provided data on safe levels of AgNP and AuNP dose levels. The present study provides an indication on systemic toxicity of these treatments, if any, expressed in the blood biochemistry and hematology.

## 2. Materials and Methods

### 2.1. Silver and Gold Nanoparticles

The colloidal AgNPs (CAS No. 7440-22-4) were purchased from ABC Nanotech (Daejeon, Korea) with at least 99.98% purity. The synthesis of these nanoparticles was based on an inductive coupled plasma (ICP) method, using silver wire as a precursor, where the resulting AgNPs were immediately stabilized using 0.9% citrate. The detailed synthesis process has already been described [8]. The percentage of Ag ions in the silver nanoparticle preparations was determined by centrifugation, through a cellulose filter with a nominal cutoff value of 3 kDa (Ultra-4, Amicon, Millipore, Germany). The total Ag content in the unfiltered AgNP suspensions, as well as their respective filtrates, was measured using ICP-MS. The percentage of soluble Ag^+^ in the AgNP preparations was calculated by dividing the Ag content in the filtrates, by the Ag^+^ content in the unfiltered AgNP suspensions, which was then multiplied by 100 [5]. The percentage of Ag ions in the 10 nm silver nanoparticles was 0.002% [7].

The AuNPs in a 1% citrate solution were provided by MINTEK (Randburg, South Africa) and have already been recognized as a reference sample (NM-330, TMU14G, 100 mg/liter) by the OECD Working Party of the Manufactured Nanomaterials (WPMN) safety testing sponsorship program [9]. The cumulative median diameter (CMD) and geometric standard deviation (GSD) of the AuNP in the 1% citrate solution were analyzed using transmission electron microscopy. The percentage of Au ions in the AuNP preparations was determined by centrifugation through a cellulose filter with a nominal cutoff value of 3 kDa (Ultra-4, Amicon, Millipore, Germany), as described above for AgNP. The percentage of Au ions in the 12.8 nm gold nanoparticles was 0.0008% [7].

### 2.2. Transmission Electron Microscopy

A transmission electron microscope equipped with an energy-dispersive X-ray analyzer (TEM-EDX) was used to measure the AgNP and AuNP based on National Institute for Occupational Safety and Health [10] analytical method 7402. The AgNP and AuNP and AgNP/AuNP mixture were analyzed in the presence of 10% serum and without serum, both in saline. The AgNP and AuNP were mounted on a TEM grid (copper grid) and visualized under a field emission-transmission electron microscope (FE-TEM, JEM2100F, JEOL, Japan). The particles were measured at a magnification of 100,000 and the AgNP and AuNP were analyzed using an energy-dispersive X-ray spectrometer (EDS, TM200, Oxford, UK) at an accelerating voltage of 75 kV.

### 2.3. Animals

Six-week-old male specific-pathogen-free (SPF) Sprague-Dawley rats (60 rats) were purchased from OrientBio (Seongnam, Korea) and were acclimated for 1 week before initiating the intravenous injection. During the acclimation and experimental periods, the rats were housed in polycarbonate cages (maximum of 3 rats per cage), which were installed in individually ventilated cage racks. The rats were kept under a controlled temperature (23 ± 2°C) and humidity (55 ± 7%) and a 12-h light/dark cycle. The rats were fed a rodent diet (Harlan Teklad, Dooyeol Biotech, Seoul, Korea) and provided with filtered water* ad libitum*. Body weights were 258.12 ± 1.94 g when nanoparticle (NP) administration commenced. Animals were divided into 7 groups (as shown in Table 1), i.e., control, AgNP (with a low dose of 10 *μ*g/kg/day and a high dose of 100 *μ*g/kg/day), AuNP (with a low dose of 10 *μ*g/kg/day and a high dose of 100 *μ*g/kg/day), and mixed AgNP/AuNP (with a low dose of 10/10 *μ*g/kg/day and a high dose of 100/100 *μ*g/kg/day). The doses were based on the blood concentration of silver after 90 days of oral administration, showing 87-419 *μ*g/g of silver in the blood [6].

The AgNP, AuNP, and control vehicle (0.9% citrate) in a 1 ml final volume, separately or in a mixture, were injected using a 1 ml syringe into the caudal vein of the rats once a day, five times per week, for 4 weeks. The nanoparticles were prepared freshly every day based on the rat body weights and used immediately. The administration of the AgNP, AuNP, and AgNP/AuNP mixture was ceased after 4 weeks and the rats were allowed to recover for another 4 weeks. A group of animals were sacrificed at the end of the injection period and an additional group also after the 4-week recovery period in order to investigate the clearance of the tissue-accumulated silver and gold. The experiment was approved by the KCL Institutional Animal Care and Use Committee according to the Korean Animal Care Act.

### 2.4. Biochemical and Hematological Evaluation

Food was withheld for 24 h before necropsy at the conclusion of the 4-week intravenous administration and after the 4-week recovery period. The rats were anesthetized with pentobarbital. Blood was then drawn from the abdominal aorta, collected in heparinized vacutainers, and analyzed for ALB (albumin), ALP (alkaline phosphatase), Ca (calcium), CHO (cholesterol), CRE (creatinine), GGTP (gamma-glutamyl transpeptidase), ALT (Alanine aminotransferase), GLU (glucose), GOT (glutamic oxaloacetic transaminase), GPT (glutamic pyruvic transaminase), IP (inorganic phosphorus), LDH (lactate dehydrogenase), Mg (magnesium), TP (total protein), UA (uric acid), BUN (blood urea nitrogen), TBIL (total bilirubin), CK (creatine phosphokinase), Na (sodium), K (potassium), Cl (chloride), TG (triglyceride), and A/G (ratio of albumin to globulin) using a biochemical blood analyzer (Hitachi 7180, Hitachi, Japan). The WBC (white blood cell count), RBC (red blood cell count), Hb (hemoglobin concentration), HCT (hematocrit), MCV (mean corpuscular volume), MCH (mean corpuscular hemoglobin), MCHC (mean corpuscular hemoglobin concentration), RDW (red cell distribution width), PLT (platelet count), MPV (mean platelet volume), NE# (number of neutrophils), NE% (percent of neutrophils), LY# (number of lymphocytes), LY% (percent of lymphocytes), MO# (number of monocytes), MO% (percent of monocytes), EO# (number of eosinophils), EO% (percent of eosinophils), BA# (number of basophils), and BA% (percent of basophils) were also analyzed using a blood cell counter (Hemavet 0950, CDC Technology, USA).

### 2.5. Coagulation Time Measurement

Following the blood collection, PT and APTT were measured by a coagulation time measurement device (ACL 7000, Instrumentation Laboratory, Bedford, MA).

### 2.6. Organ Weight Measurement

After blood collection, the rats were sacrificed by cervical dislocation. The testes, heart, thymus, lungs, spleen, liver, kidneys, and brain were all carefully removed. These organs were then weighed and fixed in a 10% formalin solution containing neutral phosphate-buffered saline. The data were expressed as relative weight (organ weight/body weight x 100).

### 2.7. Statistical Analysis

The statistical analysis was performed using SPSS (Version 19). The statistical evaluation was performed using an analysis of variance (ANOVA) following multiple comparison tests using Dunnett T3 method. The level of statistical significance was set at* p* < 0.05 and* p* < 0.01.

## 3. Results

### 3.1. Nanoparticle Size Distribution

The CMD and the GSD for the silver nanoparticles as measured by TEM were 10 nm and 1.28 nm, respectively, with a log-normal distribution between 6.4 and 13.5 nm. In contrast, the CMD and GSD of the gold analyzed TEM were 12.8 nm and 1.14, respectively, with log-normal distribution between 9 and 21. The difference in the average size was not statistically significant. A TEM image of the AgNP/AuNP mixture showed an agglomerated form; however, the particles in the mixture were well dispersed in the presence of the 10% serum in saline [7].

### 3.2. Animal Observation, Food Consumption, and Body Effects

No distinctive toxic effects from the AgNP or AuNP injections were observed during the administration and recovery period. Also, there were no significant differences in food consumption between the treated and control groups (data not shown). Likewise, there were no significant dose-related body weight changes during and after the AgNP, AuNP, or a combined administration [7]. Generally, organ weight did not show any significant changes during AgNP, AuNP, and AgNP/AuNP mix administration (Table 2). The exception, however, was a significant increase (P<0.05) in the left lung weight in the silver low- and high-dose group, when compared to the control group. In addition, a significant decrease (P<0.05) in the high-dose AgNP/AuNP mixture in the recovery group, when compared to control, was observed (Table 3).

### 3.3. Effects on Hematology and Blood Biochemistry

There were no significant dose-related changes in the hematology values for the rats (Tables 4 and 5). After the 4-week administration period of AgNP and AuNP, separately or in a mixture, the percent of eosinophil increased significantly (P<0.05) in the low dose of AgNP, whereas MCV decreased significantly (P<0.05) in the low dose of AuNP, when compared to the control.

The number of lymphocytes decreased significantly in the high-dose AgNP/AuNP mix while the MPV decreased significantly (P<0.05) in the low-dose AgNP/AuNP mixture when compared to the control. In the recovery group, the percent of neutrophils and MPV decreased significantly (P<0.05) in the high-dose AgNP/AuNP mixture when compared to the control. Sodium concentration was decreased significantly (P<0.05) after the 4-week treatment in the low- and high-dose groups of both AgNP and AuNP, as well as in the AgNP/AuNP mixture groups, when compared to the control. After the 4-week recovery period, the level of glucose increased significantly in the low- and high dose of both AgNP and AuNP groups, when compared to the control group (Tables 6 and 7).

### 3.4. Effect of AgNP and AuNP Injection on Blood Coagulation

Since a large number of nanoparticles were repeatedly injected into the blood stream, blood coagulation may be induced and, therefore, coagulation by nanoparticle injection was evaluated. Coagulation time in terms of the APTT and PT did not show any significant changes, when compared to the control group (Table 8).

## 4. Discussion

In this paper, we have investigated the effect of subacute injection of AgNP and AuNP and AgNP/AuNP mixture to blood biochemistry, hematology, and platelet coagulation. Within the dose range of our administration, the effects on the blood biochemistry and hematology were minimal and most of them were not dose-dependent. In addition, there was no significant effect on platelet coagulation after subacute injection of AgNP, AuNP, and AgNP/AuNP mixture. Therefore, the injection dose of 100 *μ*g/kg body weight would be safe dose for subacute treatment of AgNP or AuNP.

AuNPs are known to enter human cells* in vitro* without cytotoxic effects [11].* In vivo* short-term studies with different size ranges of AuNPs could also confirm no cytotoxic effects of these nanoparticles. For example, intravenous injection of small PEG-coated AuNP (4 or 13 nm) to mice, produced high levels of small size Au in the liver and spleen that peaked after 7 days and in the mesenteric lymph nodes after 1 month with no treatment related histopathological lesions. Au could be also detected in blood of these animals for 24 hr but were cleared after 7 days [12]. Likewise, after intravenous injection of various AuNPs (10, 50, 100, and 250 nm) to rats at concentrations ranging from 77 to 108 *μ*g/rat 24 hours after dosing, the majority of AuNPs were present in the liver and spleen. The 10 nm AuNPs were also present in various organs in the order of blood, liver, spleen, kidney, testis, thymus, heart, lung, and brain and, once again, with no apparent toxicity [13].

Additional studies were also conducted where a single intravenous injection of gold nanoparticles (20 nm) at 10 *μ*g/kg b.w. showed that the Au rapidly and consistently accumulated in liver and spleen throughout the entire timeframe of the study (2 months after injection). Significant accumulation of Au in the kidneys and testis could also be seen from 1-month after injection, where a decrease in Au level was observed in urine and feces. Moreover, a significant increase of Au in the blood could be seen after 2 months of injection, coinciding with the delayed accumulation in the kidneys. Although microarray results of liver and spleen showed significant effects on genes related to detoxification, lipid metabolism, cell cycle, defense response, and circadian rhythm, no apparent toxicity was reported [14]. Finally, injection of 13.5 nm AuNP at different concentrations (137.5-2200 *μ*g/kg) over 14-28 days showed that AuNP at low concentrations did not cause any appreciable toxicity and no vascular or behavioral reactions were not observed. Necropsy did not show any macroscopic organ changes in the six groups, although the injection of gold nanoparticles caused transient reversible changes in body weight of the mice within 10–14 days after injection. [15].

On the other hand, short-term studies with AgNPs could show some adverse effects. For example, a subacute (28 days) intravenous injection of AgNP (20 and 100 nm) to rats showed growth retardation with 20 nm and 100 nm AgNP treatment [16]. There was also a severe increase in spleen size and weight with increase in T and B cell numbers. Clinical chemistry indicated liver damage (increased alkaline phosphatase (ALP), ALT, and aspartate aminotransferase (AST)) and hematology showed a decrease in several red blood cell parameters. Complete suppression of the natural killer cell activity in the spleen at high doses was noted. In addition, other immune parameters were affected at these high doses such as decreased interferon-*γ* and interleukin- (IL-) 10 production by concanavalin-A stimulated spleen cells, increased IL-1ß and decreased IL-6, IL-10, and TNF-*α* production by lipopolysaccharide stimulated spleen cells, increase in serum IgM and IgE, and increase in blood neutrophilic granulocytes. Suggested critical dose was 0.37 mg/kg b.w. with immune system as being the most sensitive one [16].

After injection of AgNP (average 15 nm) at 7.5, 30, or 120 mg/kg body weight in mice, no obvious acute toxicity was observed except inflammatory reactions in lung and liver at the 120 mg/kg dose level [17]. Intravenous injection of four different doses of AgNP (15-40 nm) at 4, 10, 20, and 40 mg/kg to rats increased liver function enzymes such as alanine ALT, AST, ALP, gamma-glutamyl transpeptidase (GGTP), and bilirubin, significantly at the 40 mg/kg dose. Reactive Oxygen Species (ROS) in blood serum increased in the high-dose group, indicating that AgNP doses less than 10 mg/kg are safe for biomedical application but at high doses (> 20 mg/kg) are toxic [18].

Since major route of biomedical application of these AgNP and AuNP would be intravenous injection, ensuring data on systemic toxicity, toxicokinetics, and safe dose levels of these particles will be very valuable. Subacute toxicity studies will therefore be of relevance to calculate such safe dose levels. However, very few such studies were conducted in the literature. For example, a subchronic silver exposure to oral and inhalation studies to rats indicated that LOAEL (lowest observed adverse effect level) was 125 mg/kg for oral intake [6] and 515 *μ*g/m^3^ for inhalation [4]. At this LOAEL, liver toxicity was observed at the blood concentrations of silver of 191 ng/g for oral and 4.31 ng/g of tissue for inhalation exposure. In the present coexposure study of AgNP and AuNP, highest blood concentration of Ag was 15.97 ng/g of tissue after 28 days of 100 *μ*g/kg of AgNP intravenous administration without apparent liver toxicity. These results suggest that route of exposure may influence the toxicity of AgNP, which are different among intravenous injection or oral gavage or inhalation, thus emphasizing the observation reported earlier that tail vein injection of AuNP showed lower toxicity than oral or intraperitoneal administration [15].

Our previous publication [7] suggested that the injected AgNP and AuNP circulated in the blood mainly in particulate forms; thus the possibility that these nanoparticles may have interacted with blood components and cells to induce coagulative reaction may have existed. Earlier epidemiological studies suggested that air pollutants, for instance, particulate matter (PM) or ultrafine particles, are responsible for decreased as well as increased coagulation [19–21]. In contrast, animal exposure studies to particles of PM 2.5 did not show significant adverse effects on blood coagulation [22]. Although nanoparticle exposure has been suspected to cause blood coagulation in animal exposure studies, there were no consistent results for the induction of blood coagulation due to nanoparticle exposure. For example, exposure to amorphous silica nanoparticles (SiNPs) increased the activation of coagulation cascade by adsorbing and stimulating of intrinsic pathway coagulation factors. This effect resulted in shortening of coagulation time in APTT and PT tests, as well as in the increasing of factor X activation by RVV (Russell viper venom) in blood plasma but not in the sample with removed factors XI and XII. SiNPs did not induce platelet aggregation in platelet rich plasma but changed the shape and granularity of resting platelets and inhibited their aggregation [23]. On the other hand, subacute inhalation exposure to synthetic amorphous SiNPs up to 5.386 mg/m^3^ showed no significant differences in the blood coagulation time such as PT and APTT between control and SiNPs-exposed groups during all recovery periods [24].

In AgNP exposure studies, high dose (1000 mg/kg) of AgNP induced shortening of APPT after 28 days of oral exposure to AgNP in female rats [3]. High concentration (515 *μ*g/m^3^) of AgNP subchronic inhalation exposure increased the percent of aggregation in female rats when compared with the control [4]. This high dose or concentration had been shown to induce inflammatory reaction with hepatotoxicity in oral administration and hepatotoxicity and lung inflammation after inhalation exposure. Therefore, toxic dose might be implicated in the blood coagulation. Contrary to these observations, in our study with AgNP and AuNP or coadministration of AgNP/AuNP with maximum of 100 *μ*g/kg, each did not change PT or APPT with no changes of hepatoxicity markers or lung inflammation. In addition to dose, route of exposure and presence of inflammatory response may determine the effect of administered nanoparticles on blood coagulation parameters.

Taken together, subacute intravenous injection of AgNP or AuNP and their mixture did not result in dose-dependent changes in the hematology and blood biochemical values and blood coagulation time in terms of APTT and PT. The subacute injection of AuNP and AgNP or and their mixture did not induce any noticeable systemic toxicity.

## Figures and Tables

**Table 1:** Number of animals per dose groups for AgNP, AuNP, and AgNP/AuNP mixture administration and recovery.

Dose group	Control	AgNP		AuNP		AgNP/AuNP mix	
		Low	High	Low	High	Low^a^	High^b^
Group	G1	G2	G3	G4	G5	G6	G7

Administration	5	5	5	5	5	5	5

Recovery	3	3	3	3	3	3	3

AgNP, silver nanoparticles; AuNP, gold nanoparticles; Low, 10 *μ*g/kg/day; High, 100 *μ*g/kg/day; Low^a^, AgNP 10 *μ*g /kg/day + AuNP 10 *μ*g/kg/day; High^b^, AgNP 100 *μ*g/kg/day + AuNP 100 *μ*g/kg/day.

**Table 2:** Relative organ weights of male rats in after 4 weeks of AgNP, AuNP, and AgNP/AuNP mix administration (n-5).

Dose (*μ*g/kg)	G1	G2	G3	G4	G5	G6	G7
	Control	Silver 10	Silver 100	Gold 10	Gold 100	Mix 10	Mix 100
BODY WEIGHT (g)	368.11±8.25	356.88±8.49	378.02±18.05	374.50±13.78	400.18±13.50	378.19±6.87	381.78±14.58

TESTIS (LEFT)	0.48±0.004	0.46±0.03	0.45±0.03	0.43±0.02	0.45±0.01	0.44±0.02	0.47±0.02

TESTIS (RIGHT)	0.48±0.01	0.46±0.03	0.45±0.03	0.43±0.02	0.44±0.01	0.44±0.02	0.47±0.02

KIDNEY (LEFT)	0.37±0.02	0.37±0.02	0.37±0.01	0.36±0.01	0.37±0.01	0.38±0.02	0.36±0.01

KIDNEY (RIGHT)	0.37±0.02	0.38±0.02	0.37±0,01	0.37±0.01	0.36±001	0.36±0.01	0.37±0.003

SPLEEN	0.24±0.04	0.19±0.01	0.20±0.01	0.20±0.01	0.27±0.02	0.21±0.01	0.21±0.01

LIVER	2.99±0.05	2.79±0.08	2.90±0.12	2.78±0.08	2.97±0.04	2.75±0.09	2.71±0.03

THYMUS	0.12±0.01	0.10±0.004	0.10±0.01	0.12±0.01	0.13±0.02	0.14±0.01	0.11±0.01

HEART	0.33±0.02	0.36±0,01	0.33±0.01	0.34±0.01	0.33±0.01	0.34±0.02	0.33±0.01

LUNG (LEFT)	0.12±0,005	0.13±0.01	0.12±0.004	0.13±0.01	0.13±0.002	0.13±0.004	0.13±0.002

LUNG (RIGHT)	0.22±0.01	0.24±0.02	0.23±0.01	0.25±0.01	0.25±0.01	0.25±0.01	0.24±0.004

BRAIN	0.54±0.01	0.57±0.02	0.53±0.03	0.55±0.02	0.53±0.02	0.53±0.01	0.53±0.02

*∗*, p<0.05, comparing with control group; relative organ weight = organ weight/body weight x 100.

**Table 3:** Relative organ weights of male rats after 4 weeks of recovery (n=3).

Dose (*μ*g/kg)	G1	G2	G3	G4	G5	G6	G7
	Control	Silver 10	Silver 100	Gold 10	Gold 100	Mix 10	Mix 100
BODY WEIGHT (g)	492.75±7.03	488.05±20.72	515.00±27.76	470.37±17.00	509.79±23.61	516.41±30.25	515.12±10.65

TESTIS (LEFT)	0.37±0.02	0.36±0.01	0.37±0.03	0.41±0.01	0.35±0.02	0.33±0.03	0.33±0.005

TESTIS (RIGHT)	0.37±0.02	0.36±0.01	0.36±0.02	0.41±0.01	0.35±0.01	0.34±0.03	0.33±0.01

KIDNEY (LEFT)	0.31±0.01	0.34±0.01	0.32±0.01	0.30±0.02	0.30±0.01	0.29±0.01	0.29±0.02

KIDNEY (RIGHT)	0.32±0.01	0.35±0.02	0.33±0.02	0.32±0.01	0.29±0.01	0.29±0.01	0.29±0.02

SPLEEN	0.19±0.01	0.20±0.01	0.17±0.01	0.21±0.01	0.20±0.02	0.18±0.16	0.18±0.03

LIVER	2.78±0.16	2.86±0.18	2.68±0.15	2.73±0.16	2.69±0.08	2.73±0.01	2.53±0.07

THYMUS	0.06±0.005	0.08±0.003	0.08±0.005	0.08±0.01	0.07±0.01	0.07±0.01	0.08±0.01

HEART	0.30±0.02	0.32±0.01	0.31±0.02	0.31±0.02	0.29±0.01	0.31±0.01	0.28±0.01

LUNG (LEFT)	0.10±0.003	0.11±0.01*∗*	0.11±0.003	0.10±0.002	0.10±0.004	0.10±0.01	0.09±0.003

LUNG (RIGHT)	0.18±0.01	0.25±0.04	0.21±0.01	0.19±0.01	0.19±0.01	0.18±0.01	0.17±0.01

BRAIN	0.45±0.02	0.46±0.02	0.42±0.02	0.43±0.01	0.42±0.02	0.39±0.03*∗*	0.39±0.01*∗*

*∗*, p<0.05, comparing with control group; relative organ weight = organ weight/body weight x 100.

**Table 4:** Hematological values of male rats after 4 weeks of AgNP, AuNP, and AgNP/AuNP mix administration (n-5).

Dose (*μ*g/kg)	G1	G2	G3	G4	G5	G6	G7
	Control	Silver 10	Silver 100	Gold 10	Gold 100	Mix 10	Mix 100
WBC^1^ (K/*μ*L)	6.96±0.52	6.30±0.46	7.06±0.75	6.27±1.25	8.01±1.02	8.51±0.37	5.15±0.56

NE^2^ (K/*μ*L)	1.14±0.14	1.35±0.23	1.56±0.35	0.88±0.18	1.01±0.17	1.13±0.27	0.86±0.17

LY^3^ (K/*μ*L)	5.61±0.52	4.71±0.27	5.28±0.62	5.14±1.04	6.69±0.91	7.13±0.50	4.11±0.43^a^

MO^4^ (K/*μ*L)	0.09±0.01	0.11±0.03	0.11±0.02	0.10±0.02	0.18±0.03	0.13±0.03	0.07±0.01

EO^5^ (K/*μ*L)	0.06±0.01	0.05±0.01	0.08±0.01	0.08±0.01	0.10±0.02	0.09±0.01	0.08±0.01

BA^6^ (K/*μ*L)	0.01±0.002	0.01±0.002	0.004±0.002	0.004±0.002	0.002±0.002	0.01±0.002	0.01±0.002

NE^7^ (%)	16.86±2.69	21.06±2.79	21.84±3.33	14.30±1.78	12.82±1.46	13.48±3.30	16.40±2.36

LY^8^ (%)	80.08±2.53	75.30±3.00	74.88±3.45	82.02±1.92	83.22±1.61	83.56±3.30	80.08±2.12

MO^9^ (%)	1.30±0.16	1.72±0.25	1.54±0.28	1.46±0.19	2.30±0.35	1.52±0.45	1.42±0.23

EO^10^ (%)	0.82±0.10^b^	0.86±0.14^b^	1.24±0.20	1.40±0.19	1.34±0.19	1.10±0.12	1.50±0.17

BA^11^ (%)	0.06±0.02	0.08±0.04	0.06±0.02	0.60±0.02	0.06±0.02	0.06±0.02	0.10±0.03

RBC^12^ (M/*μ*L)	7.85±0.34	7.89±0.18	8.28±0.11	8.16±0.25	8.28±0.29	8.11±0.14	8.11±0.19

Hb^13^ (g/dL)	15.12±0.51	15.36±0.32	15.74±0.21	15.10±0.34	15.48±0.36	15.74±0.27	15.56±0.26

HCT^14^ (%)	46.02±1.32	47.12±1.17	47.48±0.59	45.72±1.15	46.58±1.10	47.16±0.73	47.30±0.69

MCV^15^ (fL)	58.82±1.02	59.68±0.57	57.44±1.22	56.12±0.91	56.36±0.69	58.20±0.29	58.40±0.70

MCH^16^ (pg)	19.30±0.30	19.46±0.17	19.04±0.44	18.52±0.39	18.72±0.23	19.42±0.07	19.18±0.19

MCHC^17^ (g/dL)	32.84±0.31	32.62±0.24	33.12±0.12	32.98±0.17	33.22±0.13	33.36±0.27	32.90±0.16

RDW^18^ (% )	11.60±0.60	11.78±0.32	11.42±0.24	11.50±0.22	11.80±0.34	11.60±0.45	11.60±0.32

PLT^19^ (K/*μ*L)	1176.00±39.04	1123.00±49.29	1053.60±56.59	1167.40±74.05	1210.80±71.8	1151.60±49.26	1094.60±23.74

MPV^20^ (fL)	10.64±0.56	11.64±0.19	11.24±0.27	9.44±1.07^d^	10.02±0.38	9.16±0.44^d^	10.58±0.26

1, white blood cell; 2, neutrophils; 3, lymphocyte; 4, monocyte; 5, eosinophil; 6, basophil; 7, percent of neutrophils; 8, percent of lymphocyte; 9, percent of monocyte; 10, percent of eosinophil; 11, percent of basophil; 12, red blood cell; 13, hemoglobin; 14, hematocrit; 15, mean corpuscular volume; 16, mean corpuscular hemoglobin; 17, mean corpuscular hemoglobin concentration; 18, red cell distribution width; 19, platelet; 20, mean platelet volume; a: p<0.05, compared with G5 and G6 group; b: p<0.05, compared with G4 and G7 group; c: p<0.05, compared with G2 group; d: p<0.05, compared with G3 and G2 group.

**Table 5:** Hematological values of male rats in after 4 weeks of recovery (n=3).

Dose (*µ*g/kg)	G1	G2	G3	G4	G5	G6	G7
	Control	Silver 10	Silver 100	Gold 10	Gold 100	Mix 10	Mix 100
WBC^1^ (K/*μ*L)	5.90±1.17	5.11±0.42	7.38±0.29	5.48±1.37	7.07±0.72	6.55±0.94	7.95±1.05

NE^2^ (K/*μ*L)	0.93±0.15	0.95±0.05	1.44±0.09	1.21±0.24	0.82±0.09	0.99±0.20	0.79±0.13

LY^3^ (K/*μ*L)	4.84±1.17	3.98±0.42	5.77±0.50	4.13±1.14	5.96±0.58	5.27±0.64	6.89±1.07

MO^4^ (K/*μ*L)	0.05±0.01	0.08±0.02	0.08±0.03	0.03±0.01	0.17±0.06	0.17±0.08	0.14±0.04

EO^5^ (K/*μ*L)	0.07±0.01	0.10±0.03	0.08±0.01	0.09±0.01	0.11±0.02	0.10±0.02	0.12±0.01

BA^6^ (K/*μ*L)	0.00±0.00	0.00±0.00	0.00±0.00	0.003±0.003	0.00±0.00	0.003±0.003	0.003±0.003

NE^7^ (%)	16.87±3.96	18.90±2.35	19.70±2.10	22.67±2.38	11.63±0.87	14.83±0.90	10.20±2.31^a^

LY^8^ (%)	80.97±3.70	77.63±1.64	77.90±2.50	74.73±2.55	84.33±1.05	81.00±1.85	86.20±2.95

MO^9^ (%)	0.90±0.15	1.53±0.35	1.20±0.47	0.67±0.07	2.23±0.72	2.33±0.83	1.73±0.52

EO^10^ (%)	1.17±0.07	1.80±0.40	1.10±0.15	1.80±0.26	1.63±0.27	1.53±0.12	1.60±0.17

BA^11^ (%)	0.00±0.00	0.03±0.03	0.03±0.03	0.03±0.03	0.00±0.00	0.07±0.03	0.03±0.03

RBC^12^ (M/*μ*L)	8.29±0.10	8.16±0.25	8.05±0.29	8.40±0.23	8.25±0.26	8.11±0.28	8.54±0.06

Hb^13^ (g/dL)	15.23±0.33	15.30±0.53	15.30±0.15	14.93±0.20	15.70±0.46	15.40±0.25	16.13±0.24

HCT^14^ (%)	44.50±0.92	45.10±1.25	45.70±0.36	45.23±0.67	46.57±1.47	47.27±0.88	48.30±1.08

MCV^15^ (fL)	53.67±0.62	55.20±0.70	56.90±2.44	53.97±1.94	56.43±0.68	58.50±3.13	56.57±1.17

MCH^16^ (pg)	18.37±0.24	18.73±0.26	19.07±0.71	17.77±0.35	18.97±0.12	19.07±0.98	18.90±0.32

MCHC^17^ (g/dL)	34.23±0.09	33.93±0.23	33.50±0.59	33.03±0.52	33.70±0.21	32.63±0.15	33.40±0.45

RDW^18^ (% )	13.07±0.77	13.27±0.35	12.67±0.23	13.17±0.52	12.67±0.20	12.83±0.07	12.50±0.36

PLT^19^ (K/*μ*L)	1010.67±54.05	1052.33±36.39	1096.00±65.82	1006.00±36.51	768.67±330.74	1068.67±72.09	991.00±130.50

MPV^20^ (fL)	10.13±0.15	9.10±0.35	7.67±1.52	10.23±0.38	10.37±0.12	8.93±0.43	6.53±0.66^b^

1, white blood cell; 2, neutrophils; 3, lymphocyte; 4, monocyte; 5, eosinophil; 6, basophil; 7, percent of neutrophils; 8, percent of lymphocyte; 9, percent of monocyte; 10, percent of eosinophil; 11, percent of basophil; 12, red blood cell; 13, hemoglobin; 14, hematocrit; 15, mean corpuscular volume; 16, mean corpuscular hemoglobin; 17, mean corpuscular hemoglobin concentration; 18, red cell distribution width; 19, platelet; 20, mean platelet volume. a: p<0.05, compared with G2, G3, and G4 group; b: p<0.01, compared with G1, G2, G4, G5, and G6 group.

**Table 6:** Serum biochemical values of male rats after 4 weeks of AgNP, AuNP, and AgNP/AuNP mix administration (n=5).

Dose (*µ*g/kg)	G1	G2	G3	G4	G5	G6	G7
	Control	Silver 10	Silver 100	Gold 10	Gold 100	Mix 10	Mix 100
ALB^1^ (g/dL)	2.16±0.04	2.22±0.05	2.16±0.02	2.16±0.05	2.08±0.05	2.08±0.04	2.10±0.03

ALP^2^ (IU/L)	460.00±45.56	542.40±44.53	619.00±93.65	553.60±54.12	455.40±38.02	551.20±32.65	516.40±20.01

CA^3^ (mg/dL)	9.34±0.07	9.14±0.08	9.50±0.25	9.14±0.11	9.38±0.17	9.38±0.13	9.68±0.35

CHO^4^ (mg/dL)	62.20±8.13	58.4010.18	67.20±8.79	67.60±3/59	62.60±7.57	51.00±2.81	54.00±3.44

CRE^5^ (mg/dL)	0.57±0.01	0.59±0.06	0.56±0.03	0.53±0.03	0.59±0.02	0.62±0.04	0.57±0.02

*γ*-GT^6^ (IU/L)	0.40±0.24	0.20±0.20	0.80±0.20	0.80±0.20	0.60±0.24	0.80±0.20	0.40±0.24

GLU^7^ (mg/dL)	123.80±7.29	149.60±9.83	164.40±10.21	138.00±8.32	152.80±10.27	164.00±15.11	139.40±3.85

AST^8^ (IU/L)	133.80±11.32	132.60±11.73	111.80±15.69	120.60±8.37	105.60±13.98	121.60±25.58	124.80±7.28

ALT^9^ (IU/L)	26.40±1.50	29.60±2.09	27.00±2.21	28.00±1.92	27.20±1;56	42.20±12.21	38.00±9.83

IP^10^ (mg/dL)	8.76±0.27	8.22±0.17	8.68±0.19	8.20±0.24	8.08±0.11	8.56±0.24	8.48±0.51

LDH^11^ (IU/L)	1587.60±176.42	1203.00±136.51	955.00±362.86	1123.40±201.42	641.00±290.21	728.40±288.19	1141.20±129.71

MG^12^ (mg/dL)	2.10±0.06	2.10±0.13	2.04±0.04	1.90±0.05	1.94±0.07	2.14±0.06	2.14±0.07

TP^13^ (g/dL)	5.38±0.08	5.54±0.09	5.50±0.14	5.54±0.09	5.46±0.09	5.44±0.11	5.36±0.14

UA^14^ (mg/dL)	1.20±0.07	1.44±0.20	1.24±0.08	1.20±0.06	1.30±0.05	1.44±0.09	1.16±0.04

BUN^15^ (mg/dL)	13.34±0.51	15.52±0.39	13.84±0.83	13.76±0.71	14.78±0.61	14.12±0.61	13.02±0.77

TBIL^16^(mg/dL)	0.05±0.02	0.05±0.01	0.07±0.01	0.05±0.01	0.05±0.01	0.06±0.003	0.05±0.01

TG^17^ (mg/dL)	38.00±7.89	24.60±6.36	43.40±16.23	29.20±6.87	28.60±4.79	30.20±5.07	20.80±3.01

CK^18^ (IU/L)	804.20±80.00	1209.80±336.07	726.20±144.96	666.20±95.67	1019.60±379.80	704.20±125.70	748.80±27.43

Na^19^ (mmol/L)	146.40±0.51	144.80±0.49	145.40±0.40^a^	145.00±0.55^a^	144.40±0.24^a^	144.40±0.51^a^	146.40±0.24

K^20^ (mmol/L)	4.40±0.22	4.70±0.11	4.68±0.10	4.80±0.06	4.60±0.08	4.72±0.11	4.60±0.12

Cl^21^ (mmol/L)	103.40±0.24	103.20±0.37	104.80±0.49	104.40±0.81	103.40±0.24	103.80±0.58	104.60±0.51

1, albumin; 2, alkaline phosphatase; 3, calcium; 4, cholesterol; 5, creatinine; 6, gamma glutamyl transpeptidase; 7, glucose; 8, aspartate amino transaminase; 9, alanine amino transaminase; 10, inorganic phosphorus; 11, lactate Dehydrogenase; 12, magnesium; 13, total protein; 14, uric acid; 15, blood urea nitrogen; 16, total bilirubin; 17, triglyceride; 18, creatine Kinase; 19, sodium; 20, potassium; 21, chloride. a: p<0.01, compared with G1 and G7 group.

**Table 7:** Serum biochemical values of male rats after 4 weeks of recovery (n=3).

Dose (*μ*g/kg)	G1	G2	G3	G4	G5	G6	G7
	Control	Silver 10	Silver 100	Gold 10	Gold 100	Mix 10	Mix 100
ALB^1^ (g/dL)	2.17±0.03	2.13±0.03	2.10±0.00	2.10±0.05	2.13±0.03	2.27±0.07	2.10±0.00

ALP^2^ (IU/L)	300.00±29.46	359.33±49.50	316.67±26.86	259.00±26.08	296.00±36.10	300.67±29.34	298.67±11.89

CA^3^ (mg/dL)	9.30±0.10	9.37±0.19	9.67±0.15	9.57±0.12	9.37±0.20	9.57±0.09	9.63±0.09

CHO^4^ (mg/dL)	75.67±11.26	61.00±11.68	64.67±9.00	60.00±4.04	73.67±17.84	90.33±6.77	68.67±5.82

CRE^5^ (mg/dL)	0.55±0.02	0.51±0.01	0.52±0.05	0.48±0.04	0.51±0.04	0.53±0.02	0.48±0.04

*γ*-GT^6^ (IU/L)	0.33±0.33	0.33±0.33	0.00±0.00	1.00±0.00	0.33±0.33	0.00±0.00	0.33±0.33

GLU^7^ (mg/dL)	131.33±4.37^a^	132.67±6.69^a^	150.67±6.17^a^	144.33±8.19^a^	137.33±6,44^a^	168.67±12.33	176.33±2.73

AST^8^ (IU/L)	185.00±24.99	164.33±10.68	195.67±45.19	130.00±7.09	160.00±9.81	180.00±21.79	100.33±15.25

ALT^9^ (IU/L)	35.67±2.33	38.67±1.20	56.00±24.58	30.67±1.33	34.67±1,20	37.67±2.19	38.33±7.13

IP^10^ (mg/dL)	6.97±0.12	7.30±0.32	7.60±0.29	7.30±	7.80±0.32	7.23±0.22	6.50±0.31

LDH^11^ (IU/L)	2007.00±466.88	1373.33±196.70	1467.67±41.00	952.00±166.73	1663.00±96.63	1650.67±458.90	202.67±41.58

MG^12^ (mg/dL)	1.00±0.00	2.00±0.00	3.00±0.00	4.00±0.00	5.00±0.00	6.00±0.00	7.00±0.00

TP^13^ (g/dL)	5.77±0.09	5.50±0.12	5.50±0.06	5.43±0.13	5.73±0.07	5.83±0.18	5.70±0.06

UA^14^ (mg/dL)	1.23±0.13	1.37±0.07	1.13±0.13	1.33±0.09	1.20±0.10	1.30±0.10	1.03±0.12

BUN^15^ (mg/dL)	16.10±0.10	15.50±0.06	17.50±1.21	14.37±1.21	17.23±0.15	18.37±1.46	14.77±0.24

TBIL^16^(mg/dL)	0.05±0.01	0.05±0.01	0.08±0.01	0.08±0.02	0.04±0.02	0.08±0.01	0.08±0.01

TG^17^ (mg/dL)	43.00±13.45	34.67±15.67	36.33±10.91	31.33±5.81	46.00±10.15	44.00±6.93	48.67±8.21

CK^18^ (IU/L)	1237.67±346.14	1299.67±114.17	1007.00±243.60	705.00±73.66	1293.33±154.34	1093.33±109.25	829.33±316.17

Na^19^ (mmol/L)	143.67±0.33	145.00±0.58	144.33±0.33	145.33±0.67	146.00±0.58	146.67±0.88	145.67±0.67

K^20^ (mmol/L)	4.77±0.15	4.80±0.06	4.67±0.12	4.47±0.12	4.93±0.13	4.70±0.10	4.33±0.12

Cl^21^ (mmol/L)	103.33±0.33	104.33±0.33	104.33±0.33	106.00±0.58	105.33±1.20	104.67±0.67	106.33±0.88

1, albumin; 2, alkaline phosphatase; 3, calcium; 4, cholesterol; 5, creatinine; 6, gamma glutamyl transpeptidase; 7, glucose; 8, aspartate amino transaminase; 9, alanine amino transaminase; 10, inorganic phosphorus; 11, lactate dehydrogenase; 12, magnesium; 13, total protein; 14, uric acid; 15, blood urea nitrogen; 16, total bilirubin; 17, triglyceride; 18, creatine Kinase; 19, sodium; 20, potassium; 21, chloride:; a: p<0.01, compared with G6 and G7 group.

**Table 8:** Blood coagulation test of male rats in injection and recovery group.

Group	APTT^†^ (mean±S.E)	PT^‡^ (mean±S.E)
Control Injection (5)	14.96 ± 1.29	14.12 ± 0.58

Control Recovery (3)	17.47 ± 1.28	15.43 ± 0.94

AgNP 10 *μ*g/kg Injection (5)	16.14 ± 0.77	14.44 ±0.29

AgNP 10 *μ*g/kg Recovery (3)	16.40 ± 0.55	15.47 ± 1.03

AgNP 100 *μ*g/kg Injection (5)	15.64 ± 0.67	14.26 ± 0.47

AgNP 100 *μ*g/kg Recovery (3)	17.50 ± 0.20	15.90 ± 0.40

AuNP 10 *μ*g/kg Injection (5)	14.14 ± 0.67	14.12 ± 0.49

AuNP 10 *μ*g/kg (3) Recovery	16.93 ± 0.29	15.70 ± 0.21

AuNP 100 *μ*g/kg Injection (5)	16.88 ± 1.14	15.20 ± 0.35

AuNP 100 *μ*g/kg Recovery (3)	14.90 ± 0.95	14.80 ± 0.67

AgNP/AuNP Mix Injection 10/10 *μ*g/kg (5)	16.40 ± 0.50	16.06 ± 0.45

AgNP/AuNP Mix recovery 10/10 *μ*g/kg (3)	16,57 ± 0.85	16.23 ± 0.17

AgNP/AuNP Mix Injection 100/100 *μ*g/kg (5)	18.04 ± 1.76	14.70 ± 0.63

AgNP/AuNP Mix Recovery 100/100 *μ*g/kg (3)	17.30 ± 1.22	17.30 ± 1.12

( ): number of animals.

†: activated partial thromboplastin time (sec).

‡: prothrombin time (sec).

## Data Availability

The data used to support the findings of this study are included within the article.

## Abbreviations

AgNP: Silver nanoparticles
AuNP: Gold nanoparticles
APTT: Active partial thromboplastin time
PT: Prothrombin time
ALB: Albumin
ALT: Alanine aminotransferase
AST: Aspartate aminotransferase
ALP: Alkaline phosphatase
Ca: Calcium
CRE: Creatinine
GLU: Glucose
GGTP: Gamma-glutamyl transpeptidase
ICP-MS: An inductive coupled plasma mass spectrophotometer
GOT: Glutamic oxaloacetic transaminase
GPT: Glutamic pyruvic transaminase
IP: Inorganic phosphorus
LDH: Lactate dehydrogenase
Mg: Magnesium
TP: Total protein
UA: Uric acid
BUN: Blood urea nitrogen
TBIL: Total bilirubin
CK: Creatine phosphokinase
Na: Sodium
K: Potassium
Cl: Chloride
TG: Triglyceride
A/G: Ratio of albumin to globulin
WBC: White blood cell count
RBC: Red blood cell count
Hb: Hemoglobin concentration
HCT: Hematocrit
MCV: Mean corpuscular volume
MCH: Mean corpuscular hemoglobin
MCHC: Mean corpuscular hemoglobin concentration
RDW: Red cell distribution width
PLT: Platelet count
MPV: Mean platelet volume
NE#: Number of neutrophils
NE%: Percent of neutrophils
LY#: Number of lymphocytes
LY%: Percent of lymphocytes
MO#: Number of monocytes
MO%: Percent of monocytes
EO#: Number of eosinophils
EO%: Percent of eosinophils
BA#: Number of basophils
BA%: Percent of basophils
CMT: Count median diameter
GSD: Geometric standard deviation
LOAEL: Lowest observed adverse effect level.
